# Computational modeling and simulation of epithelial wound closure

**DOI:** 10.1038/s41598-023-33111-4

**Published:** 2023-04-17

**Authors:** Jie Bai, Xiaowei Zeng

**Affiliations:** Department of Mechanical Engineering, University of Texas at San Antonio, One UTSA Circle, San Antonio, TX 78249 USA

**Keywords:** Biophysics, Biomedical engineering, Computational science

## Abstract

Wounds in the epithelium may lead to serious injurious events or chronic inflammatory diseases, however, multicellular organisms have the ability to self-repair wounds through the movement of epithelial cell toward the wound area. Despite intensive studies exploring the mechanism of wound closure, the role of mechanics in epithelial wound closure is still not well explained. In order to investigate the role of mechanical properties on wound closure process, a three-dimensional continuum physics-based computational model is presented in this study. The model takes into account the material property of the epithelial cell, intercellular interactions between neighboring cells at cell–cell junctions, and cell-substrate adhesion between epithelial cells and ECM. Through finite element simulation, it is found that the closure efficiency is related to the initial gap size and the intensity of lamellipodial protrusion. It is also shown that cells at the wound edge undergo higher stress compared with other cells in the epithelial monolayer, and the cellular normal stress dominates over the cellular shear stress. The model presented in this study can be employed as a numerical tool to unravel the mechanical principles behind the complex wound closure process. These results might have the potential to improve effective wound management and optimize the treatment.

## Introduction

Collective cell migration plays an important role in many biological activities such as tissue morphogenesis, wound repair, and cancer metastasis^1^. In the epithelium wound healing process, it involves the closure of epithelial gaps during which collective cell migration enables the regeneration of a functional tissue^2,3^. Rapid wound healing to restore tissue's physiological functions could prevent further damage. Many studies have been oriented on wound closure since it is important to maintain epithelial functions and homeostasis. It has been found that gap closure was mediated by two distinct mechanisms^2,4^. The first mechanism is based on the assembly and contraction of a multicellular actomyosin cable at the wound edge in a purse-string-like manner in which the driving force is provided by the contraction of the actomyosin cable around the wound^5–7^. The second mechanism is based on cell crawling mediated by lamellipodial protrusion^8–10^. The two mechanisms may have different contributions to wound closure depending on the biochemical and biophysical properties of the environment. However, it remains poorly understood how these two modes which are driven by the assembly of distinct actin networks, are regulated in wound healing process. Previous experiments have reported key factors on how the physical forces influence collective cell migration^2,11–14^. Some in vitro experiments identified that closure of large wounds is initiated by cell crawling driven by the lamellipodial protrusion, then followed by the purse-string assembly when wound sizes become smaller at the later stage of closure^14,15^. Purse-string behaves like a contractile cable that pulls the wound edge at a speed proportional to its local curvature^9^. By contrast, closure is dominated by lamellipodium-mediated cell migration in large gaps. Some experiments found that material properties such as substrate stiffness of extracellular matrices and cell stiffness affect cell morphology, proliferation, and migration^16–19^. It was also observed that intercellular adhesion also regulates the wound closure efficiency^20^. However, it remains poorly understood how the mechanical properties of cells and their interactions regulate the purse-string and cell crawling motion. Since experiments are limited in the capability of explaining the mechanical influences that are separated from biochemical processes, mathematical and computational models might better decouple these attributes.

To date, plenty of work has been done to model the collective cell migration during tissue repair and tissue morphogenesis^21–26^. Continuum models^27^ considering cell mechanical properties were able to successfully capture the collective flow and traction force observed in experiments, by modeling epithelial tissues as viscoelastic fluids^15,22^ or elastic materials^9,23,28^. However, these continuum models cannot capture the cellular scale dynamics, therefore were not able to connect individual cell properties to collective cell dynamics. On the other hand, cellular-based numerical models, including the Cellular Potts Model^29,30^, hybrid cellular Potts model^31^, particle-based models^32–34^, vertex Model^35–37^, and phase-field model^38^ were widely used to study epithelial cells with great success. However, these particle-based models cannot capture the deformation and stress in the cells since the material properties of cells were not modeled explicitly. Since the intercellular interaction at the cell–cell junction surface is not modeled directly, how an individual cell interacts with its neighboring cells to move collectively is not clear. It is very important to investigate how these mechanical cues regulate the cell motility and wound closure efficiency in response to changes in wound size and shapes during the epithelial wound closure process^39^.

To overcome these limitations, we propose a continuum physics-based computational model at the cellular level that incorporates the cell stiffness, lamellipodial protrusion, intercellular interaction, and cell-substrate adhesion to study the influence of mechanical cues on epithelial wound closure without cell division and proliferation. In this study, an interfacial interaction model based on the cohesive zone traction–separation law^40,41^ was proposed to model the intercellular interactions for the collective cell migration during the wound closure process for the first time. Using this model, we perform numerical case studies to investigate how the cell mechanical properties and mechanical interactions regulate the cell motility and wound closure efficiency during the epithelial wound closure. In particular, we find that the promotion of lamellipodial protrusion leads to a higher wound closure rate, and the stress is found to be concentrated at the wound edge. In addition, the closure time is found to vary linearly with the initial gap size. The computational model presented might give rise to a better understanding of the wound closure mechanism from the mechanical point of view.

## Methods and material

### Geometric modeling of 3D epithelial monolayer sheet

**Figure 1:**
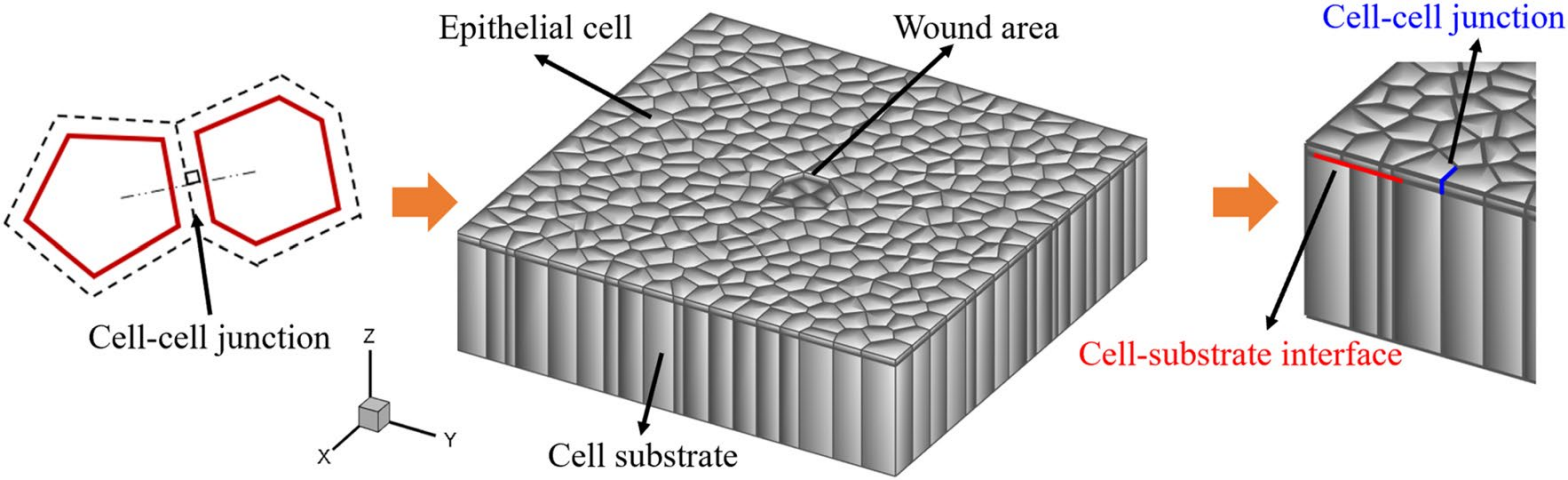
Process of generating the 3D geometry of epithelial monolayer sheet for the wound healing process. First, a 2D model of the epithelial monolayer sheet was generated via Voronoi tessellation. The wound was created by removing several cells in the middle of the sheet. The 2D model was then extruded to achieve the epithelium thickness. Eventually, an ECM substrate was built underneath the monolayer.

The thickness of the epithelial monolayer was reported between 3 μm and 15 μm^42,43^. To create the three-dimensional (3D) geometry of the model, first, a two-dimensional (2D) representation of the epithelial monolayer sheet which contains polygon-shaped epithelial cells was created using the Voronoi tessellation method^44^. The two-dimensional epithelial monolayer we created is a 400 μm × 400 μm square sheet with the 26 μm diameter of average cell size according to experimental measurements^45^. Then, the 10 nm cell–cell junctions were generated to characterize the intercelluar interaction between neighboring cells throughout the model^46^. Finally, the generated 2D model was extruded along the out of plane direction to represent the epithelial monolayer thickness. A substrate was built underneath the epithelial monolayer. The wound was created by removing a couple of cells in the middle of the epithelial sheet. The geometric configuration of the three-dimensional epithelial monolayer is shown in Fig. 1.

### Intercellular interaction modeling at the cell–cell junctions

**Figure 2:**
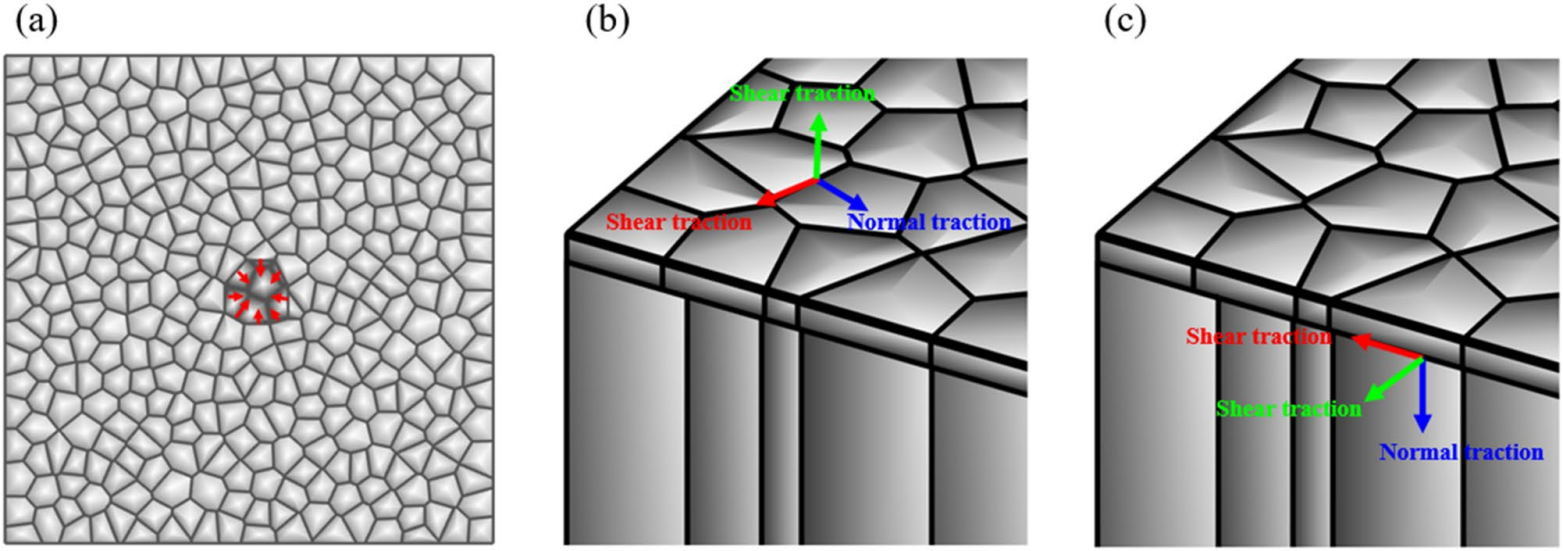
Interaction forces modeling: (**a**) Protrusion traction force at the wound edge, pointing toward the wound center; (**b**) Intercellular interaction at cell–cell junctions was decomposed into normal traction and shear traction; (**c**) Cell-substrate adhesion at the cell-substrate interface was decomposed into normal traction and shear traction.

The transmission of mechanical forces is broadly recognized to play a significant role during the cell migration process. While the motile cells move, they will interact with their neighboring cells, and the interaction force will be transmitted through cell–cell interactions at cell–cell junctions^47^. Mechanical stresses exerted at cell–cell junctions have been studied in experiments with different measurements^13,48–50^. From these experiments, the intercellular interaction within a migrating monolayer can be decomposed into normal traction ${\mathrm{T}}_{\mathrm{n}}^{\mathrm{c}-\mathrm{c}}$ that is perpendicular to the cell–cell junction and shear traction ${\mathrm{T}}_{\mathrm{t}}^{\mathrm{c}-\mathrm{c}}$ that is tangential to the cell–cell junction as shown in Fig. 2.

Some computational frameworks such as the vertex model and cellular potts model have been developed to study the cell jamming/unjamming transition^29,51^. These models incorporate the contribution of cell cortical tension and particle-based intercellular adhesion, however, they are mathematical models that could not capture the mechanical behavior of the cell tissue and intercellular interaction at the cell–cell junction surface directly. In this study, the intercellular interactions at cell–cell junctions were modeled using an interfacial interaction model which was proposed by Lin et al. to investigate the collective epithelial cell migration^41^, and it takes the following forms:

1 $${T}_{n}^{c-c}=\left\{\begin{array}{ll}{\sigma }_{c-c}\left(\frac{{d}_{n}-{\delta }_{0}}{{\delta }_{dn}-{\delta }_{0}}\right){\left[{e}^{1-\frac{{d}_{n}-{\delta }_{0}}{{\delta }_{dn}-{\delta }_{0}}}\right]}^{{q}_{n}} & {d}_{n}\le {\delta }_{dn} \\ {\sigma }_{c-c}{\left(\frac{{\delta }_{fn}-{d}_{n}}{{\delta }_{fn}-{\delta }_{dn}}\right)}^{{p}_{n}} & {\delta }_{dn}<{d}_{n}<{\delta }_{fn}\\ 0 & {d}_{n}\ge {\delta }_{fn}\end{array}\right.$$

2 $${T}_{t}^{c-c}=\left\{\begin{array}{ll}{\tau }_{c-c}\left(\frac{{d}_{t}}{{\delta }_{dt}}\right){\left[{e}^{\frac{1}{2}-\frac{{d}_{t}^{2}}{{2\delta }_{dt}^{2}}}\right]}^{{q}_{t}} & 0\le \left|{d}_{t}\right|\le {\delta }_{dt}\\ {\tau }_{c-c}\frac{{d}_{t}}{\left|{d}_{t}\right|}{\left(\frac{{\delta }_{ft}-\left|{d}_{t}\right|}{{\delta }_{ft}-{\delta }_{dt}}\right)}^{{p}_{t}}& {\delta }_{dt}\le \left|{d}_{t}\right|\le {\delta }_{ft}\\ 0 & \left|{d}_{t}\right|\ge {\delta }_{ft}\end{array}\right.$$

There are six independent parameters in the cell–cell junction surface normal direction in the presented intercellular interaction model: ${\sigma }_{c-c}$, ${\delta }_{dn}$, ${\delta }_{0}$, ${\delta }_{fn}$, ${q}_{n}$ and ${p}_{n}$, and five independent parameters in the junction surface tangential direction: ${\tau }_{c-c}$, ${\delta }_{dt}$, ${\delta }_{ft}$, ${q}_{t}$ and ${p}_{t}$. These parameters regulate different intercellular adhesion properties and behaviors. In the above equations, ${\sigma }_{c-c}$ and ${\tau }_{c-c}$ represent the value of intercellular adhesion strength in the cell–cell junction surface at the normal and the tangential directions respectively, ${d}_{n}$ and ${d}_{t}$ are the normal and the tangential surface separations respectively, ${\delta }_{0}$ represents the equilibrium distance of intercellular interactions. There are two critical intercellular interaction distance ${\delta }_{dn}$ and ${\delta }_{dt}$ along the surface normal and the surface tangential direction, respectively. When the cell–cell junction surface separation is larger than the critical intercellular interaction distance, the intercellular adhesion strength decreases as the separation increases. Here ${q}_{n}, {q}_{t}, {p}_{n}, {p}_{t}$ are the shape parameters, ${\delta }_{fn}$ and ${\delta }_{ft}$ are intercellular interaction cutoff distance along the cell–cell junction surface normal and tangential direction, respectively. This model has been employed to describe intercellular interactions in collective cell migration modeling and other biological material interface modelings^41,52^.

### Cell-substrate adhesion modeling

Cell movement also involves cell-substrate adhesion which is the attachment of a cell to the underlying extracellular matrix (ECM) substrate through mechanosensitive focal adhesion complexes of the integrin family^53^. It enables cell activity in the ECM to affect the cell shape and movement. There have been many efforts in the modeling of the cell-substrate interactions^54–57^. An exponential cohesive zone model^58^ was employed to represent the cell-substrate interfacial behavior^56^. The normal traction ${T}_{n}^{c-s}$ and shear traction ${T}_{t}^{c-s}$ of the cell-substrate adhesion take the similar expressions as the intercellular interaction stated above. Their expressions are described as the following terms:

3 $${T}_{n}^{c-s}=\left\{\begin{array}{ll}{\sigma }_{c-s}\left(\frac{{d}_{v}-{\delta }_{0}}{{\delta }_{dv}-{\delta }_{0}}\right){\left[{e}^{1-\frac{{d}_{v}-{\delta }_{0}}{{\delta }_{dv}-{\delta }_{0}}}\right]}^{{q}_{n}}& {d}_{v}\le {\delta }_{dv} \\ {\sigma }_{c-s}{\left(\frac{{\delta }_{fv}-{d}_{v}}{{\delta }_{fv}-{\delta }_{dv}}\right)}^{{p}_{n}}& {\delta }_{dv}<{d}_{v}<{\delta }_{fv}\\ 0 & {d}_{v}\ge {\delta }_{fv}\end{array}\right.$$

4 $${T}_{t}^{c-s}=\left\{\begin{array}{ll}{\tau }_{c-s}\left(\frac{{d}_{u}}{{\delta }_{du}}\right){\left[{e}^{\frac{1}{2}-\frac{{d}_{u}^{2}}{{2\delta }_{du}^{2}}}\right]}^{{q}_{t}} & 0\le \left|{d}_{u}\right|\le {\delta }_{du}\\ {\tau }_{c-s}\frac{{d}_{u}}{\left|{d}_{u}\right|}{\left(\frac{{\delta }_{fu}-\left|{d}_{u}\right|}{{\delta }_{fu}-{\delta }_{du}}\right)}^{{p}_{t}} & {\delta }_{du}\le \left|{d}_{u}\right|\le {\delta }_{fu}\\ 0 & other\end{array}\right.$$

In the above equations, ${\sigma }_{c-s}{ \, {\text{and}} \, \tau }_{c-s}$ represents the adhesion strength in the cell-substrate surface normal and tangential direction, respectively. In this study,${\tau }_{c-s}$ is set to be 5 Pa according to the previous experimental measurement^12^, ${d}_{v} \, {\text{and}} \, {d}_{u}$ are the normal and tangential separations between the cell and the substrate, respectively. The critical interaction distance ${\delta }_{dv} \, {\text{and}} \, {\delta }_{du}$ are the distance between the cell and the substrate in the normal and tangential direction when cell-substrate adhesion reaches its maximum value, and both are set to be 25 nm according to previous study^56^. The detachment distance ${\delta }_{fv} \, {\text{and}} \, {\delta }_{fu}$ are the distances between the cell and the substrate in the normal and tangential direction when cell-substrate adhesion decrease to zero. It is estimated to be 60 nm according to the reported measurements^59,60^.

### Protrusion force modeling

In this study, we considered the epithelial cell crawling to the wound area due to traction forces. The onset of the wound closure involved rapid protrusion of lamellipodia and filopodia at the wound edge toward the wound area^39^. The traction force applied on the lamellipodia is assumed to be 2 pN pointing toward the wound center, as shown in Fig. 2a. In addition, it was shown that each cell in an advancing epithelial monolayer was also involved in a global tug-of-war^12^. Therefore we apply 0–0.3 pN protrusion forces on the edges of all other cells in random directions.

### Finite element implementation

In this study, a displacement-based finite element (FE) model which is evolved from the virtual work principle was developed using our in-house FE software package written in FORTRAN language. A Galerkin weak formulation for the FE simulation can be expressed as follows:

5 $${\int _\Omega}\uprho \ddot{\mathrm{u}}\cdot\updelta \mathbf{u}\mathrm{d\Omega }= {\int_\mathrm{S_{edge}} }{\mathbf{T}}^{\mathrm{p}}\cdot\updelta \mathbf{u}\mathrm{dS}+{\int_{\mathrm{S}_{\mathrm{c-c}}} }{\mathbf{T}}^{\mathrm{c}-\mathrm{c}}\cdot\updelta \Delta \mathrm{dS}+{\int_{\mathrm{S}_{\mathrm{c-s}}}}{\mathbf{T}}^{\mathrm{c}-\mathrm{s}}\cdot\updelta \mathbf{u}\mathrm{dS}-{\int_\Omega }\mathbf{P} :\updelta \mathbf{F}\mathrm{d\Omega }$$

where $\uprho$ is the material density of the cell, $\Omega$ is the cell volume, ${\mathrm{S}}_{\mathrm{edge}}$ is the cell edge surface, ${\mathrm{S}}_{\mathrm{c}-\mathrm{c}}$ is the cell–cell junction surface, ${\mathrm{S}}_{\mathrm{c}-\mathrm{s}}$ is the cell-substrate interaction surface, ${\mathbf{T}}^{\mathrm{p}}$ is the protrusion traction vector, ${\mathbf{T}}^{\mathrm{c}-\mathrm{c}}$ and ${\mathbf{T}}^{\mathrm{c}-\mathrm{s}}$ are the intercellular interaction traction vector and the cell-substrate interaction traction vector respectively, **P** is the first Piola–Kirchhoff stress tensor, and $\mathbf{P}:\updelta \mathbf{F}= {\mathrm{P}}^{\mathrm{ij}}\updelta {\mathrm{F}}_{\mathrm{ji}}$. The discrete equations of motion can be expressed in the following forms:

6 $$\mathbf{M}\ddot{\mathrm{u}}= {\mathbf{F}}_{\mathrm{ext}}- {\mathbf{F}}_{\mathrm{int}}$$

7 $${\mathbf{F}}_{\mathrm{ext}}= {\mathbf{F}}^{\mathrm{p}}+ { {\mathbf{F}}^{\mathrm{c}-\mathrm{c}}+\mathbf{F}}^{\mathrm{c}-\mathrm{s}}$$

In the above equations, **M** is the mass matrix, ${\mathbf{F}}_{\mathrm{ext}}$ is the external force that involves the protrusion force ${\mathbf{F}}^{\mathrm{p}}$, the intercellular interaction force ${\mathbf{F}}^{\mathrm{c}-\mathrm{c}}$ and the cell-substrate adhesion force ${\mathbf{F}}^{\mathrm{c}-\mathrm{s}}$. ${\mathbf{F}}_{\mathrm{int}}$ represents the internal force resulting from the epithelial cell deformation.

### Material properties and parameters

In this study, the epithelial cell was assumed as an isotropic elastic material and Young’s modulus is set to be 0.2 kPa, and Poisson’s ratio $\nu =0.45$ according to the measurements reported in the previous experiments^61,62^. The mass density is set to be $2\times {10}^{-3} ng/{\upmu {\text{m}}}^{3}$ for individual epithelial cell based on the previous measurements^63,64^. The detailed parameters for the intercellular interaction at cell–cell junctions and cell-substrate adhesion at the cell-substrate interface are from previous studies^65–68^, and are listed in Table 1.

**Table 1:** Intercellular interaction parameters and cell-substrate interaction parameters.

${\upsigma }_{\mathrm{c}-\mathrm{c}}$	${\uptau }_{\mathrm{c}-\mathrm{c}}$	${\updelta }_{\mathrm{dn}}$	${\updelta }_{\mathrm{fn}}$	${\updelta }_{\mathrm{dt}}$	${\updelta }_{\mathrm{ft}}$	${\updelta }_{0}$	${\mathrm{q}}_{\mathrm{n}}$	${\mathrm{p}}_{\mathrm{n}}$	${\mathrm{q}}_{\mathrm{t}}$	${\mathrm{p}}_{\mathrm{t}}$
2 nN/${{\upmu {\text{m}}}}^{2}$	2 nN/${{\upmu {\text{m}}}}^{2}$	1 ${\upmu {\text{m}}}$	2 ${\upmu {\text{m}}}$	1 ${\upmu {\text{m}}}$	2 ${\upmu {\text{m}}}$	10 nm	1.0	1.0	1.0	1.0

${\upsigma }_{\mathrm{c}-\mathrm{s}}$	${\uptau }_{\mathrm{c}-\mathrm{s}}$	${\updelta }_{\mathrm{dv}}$	${\updelta }_{\mathrm{fv}}$	${\updelta }_{\mathrm{du}}$	${\updelta }_{\mathrm{fu}}$	${\updelta }_{0}$	${\mathrm{q}}_{\mathrm{n}}$	${\mathrm{p}}_{\mathrm{n}}$	${\mathrm{q}}_{\mathrm{t}}$	${\mathrm{p}}_{\mathrm{t}}$
5 N/${\mathrm{m}}^{2}$	5 N/${\mathrm{m}}^{2}$	25 n $\mathrm{m}$	60 n $\mathrm{m}$	25 n $\mathrm{m}$	60 n $\mathrm{m}$	1 nm	1.0	1.0	1.0	1.0

## Results and discussion

### Stress distribution in the epithelial monolayer during wound closure

**Figure 3:**
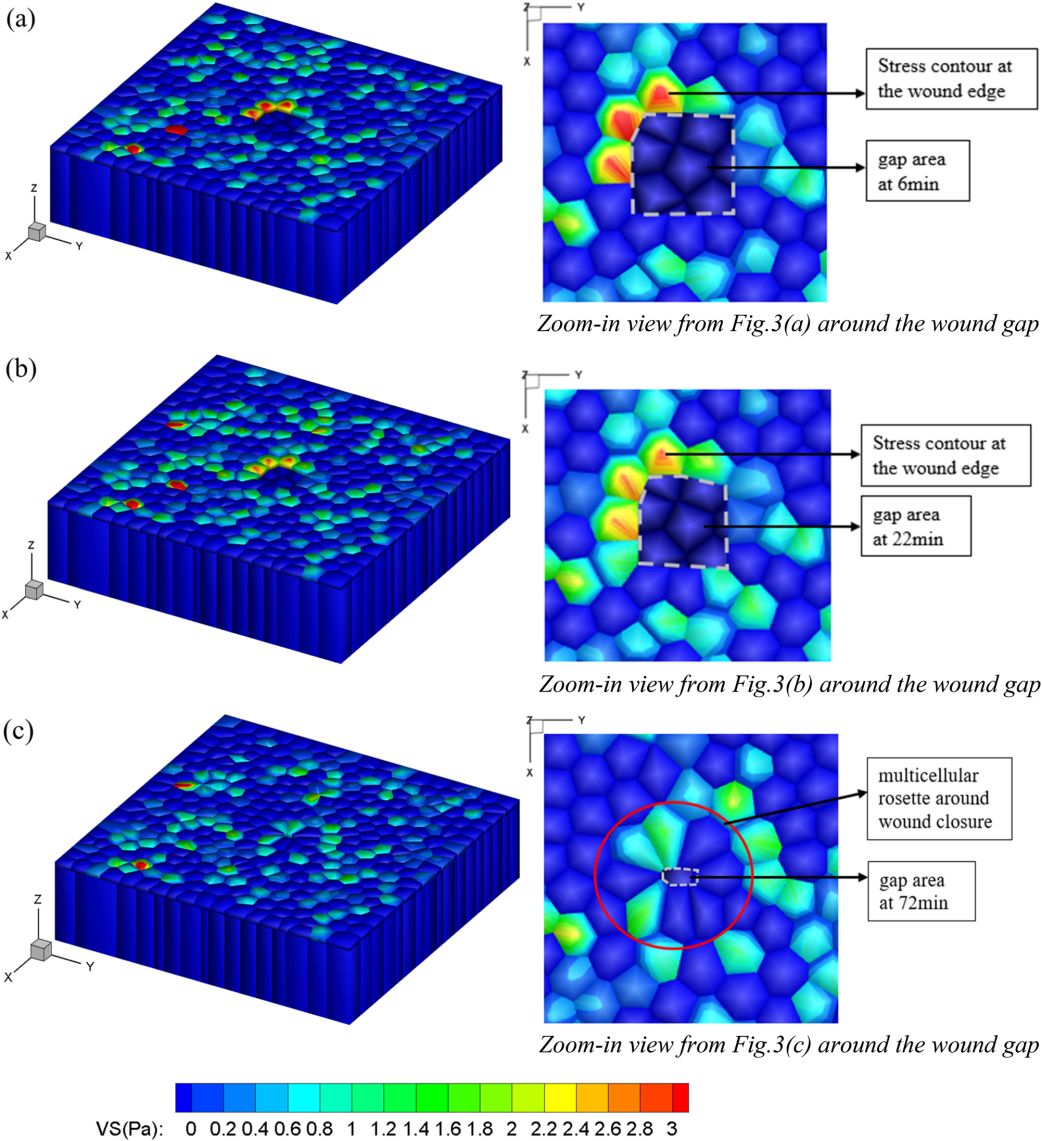
Snapshots of von-Mises stress distribution of 3D epithelial wound closure model (left) and their top-down zoom-in view of the wound area (right) during wound closure process: (**a**) t = 6 min; (**b**) t = 22 min; (**c**) t = 72 min. The color contour shows the stress distribution and its change with time during the wound closure process.

In the finite element simulation, each cell in the monolayer sheet was discretized into wedge elements to perform the computation. We plotted the von-Mises stress during the wound closure process as shown in Fig. 3 where the initial gap area is 3136 ${\upmu {\text{m}}}^{2}$. At the beginning, cells migrated toward the center of the wound area and cells were elongated along the direction of migration due to the traction force applied at the cell edges (Fig. 3). When the wound starts to close, cells typically form a rosette-like structure as shown in Fig. 3c, which was consistent with the experimental observation^2^. It can be observed that throughout the time, several neighboring junctions shrink to a single point to give rise to a multicellular rosette, as shown in Fig. 3c. The completely shrunk single-junction enabled intercalations which bring new junctional associations between previously non-neighboring cells and these new junctions always localize parallel to the closing wound^69^. The epithelial wound healing process can be seen in Supplementary Movie S1.

**Figure 4:**
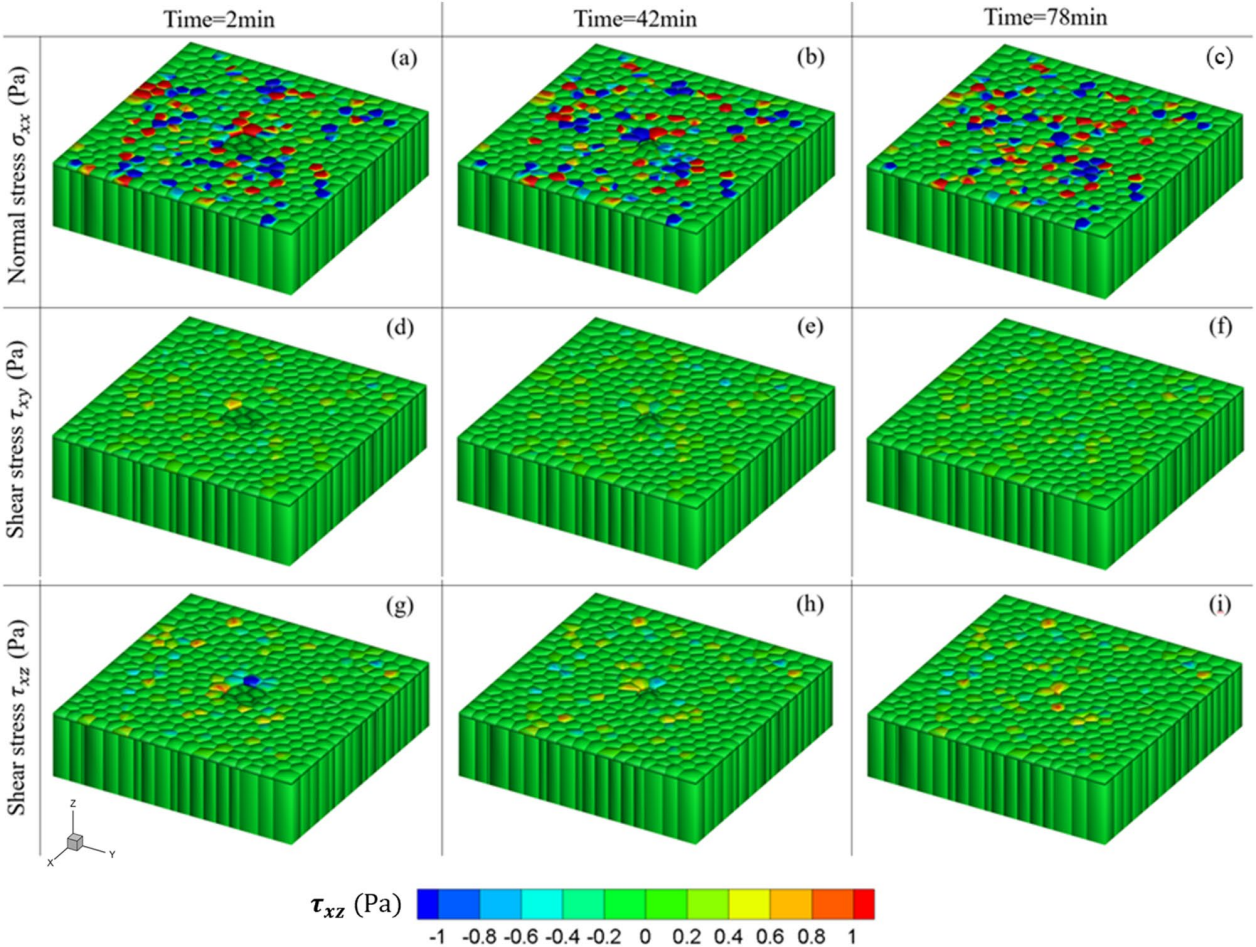
Snapshots of comparing the normal stress distribution with the shear stress distribution during the wound closure process: (**a**–**c**) show the normal stress ${{\varvec{\sigma}}}_{{\varvec{x}}{\varvec{x}}}$ at different timestep; (**d**–**f**) show the shear stress ${{\varvec{\tau}}}_{{\varvec{x}}{\varvec{y}}}$ at different timestep; (**g**–**i**) show the shear stress ${{\varvec{\tau}}}_{{\varvec{x}}{\varvec{z}}}$ at different timestep.

The wound closure response consists of cell movements toward the wound followed by the formation of multicellular actin bundles and leader cells with active actin-based protrusions^70^. One might see the stress concentration (red color region) at the wound edge at the beginning of the healing process (Fig. 3a), but the stress decreases as the wound closes (Fig. 3b,c). We also plotted the normal stress and the shear stress distribution overtime during the wound closure process as shown in Fig. 4. One might observe that the distribution of normal stress is heterogeneous compared to the other two distributions of shear stress components. The fluctuations of average normal stress occur over the whole monolayer sheet. In addition, the value of normal stress is greater than that of the shear stresses. These observations were consistent with the experimental investigation that the average local normal stress is severely heterogeneous^13^.

### Effect of the initial gap size on wound closure efficiency

**Figure 5:**
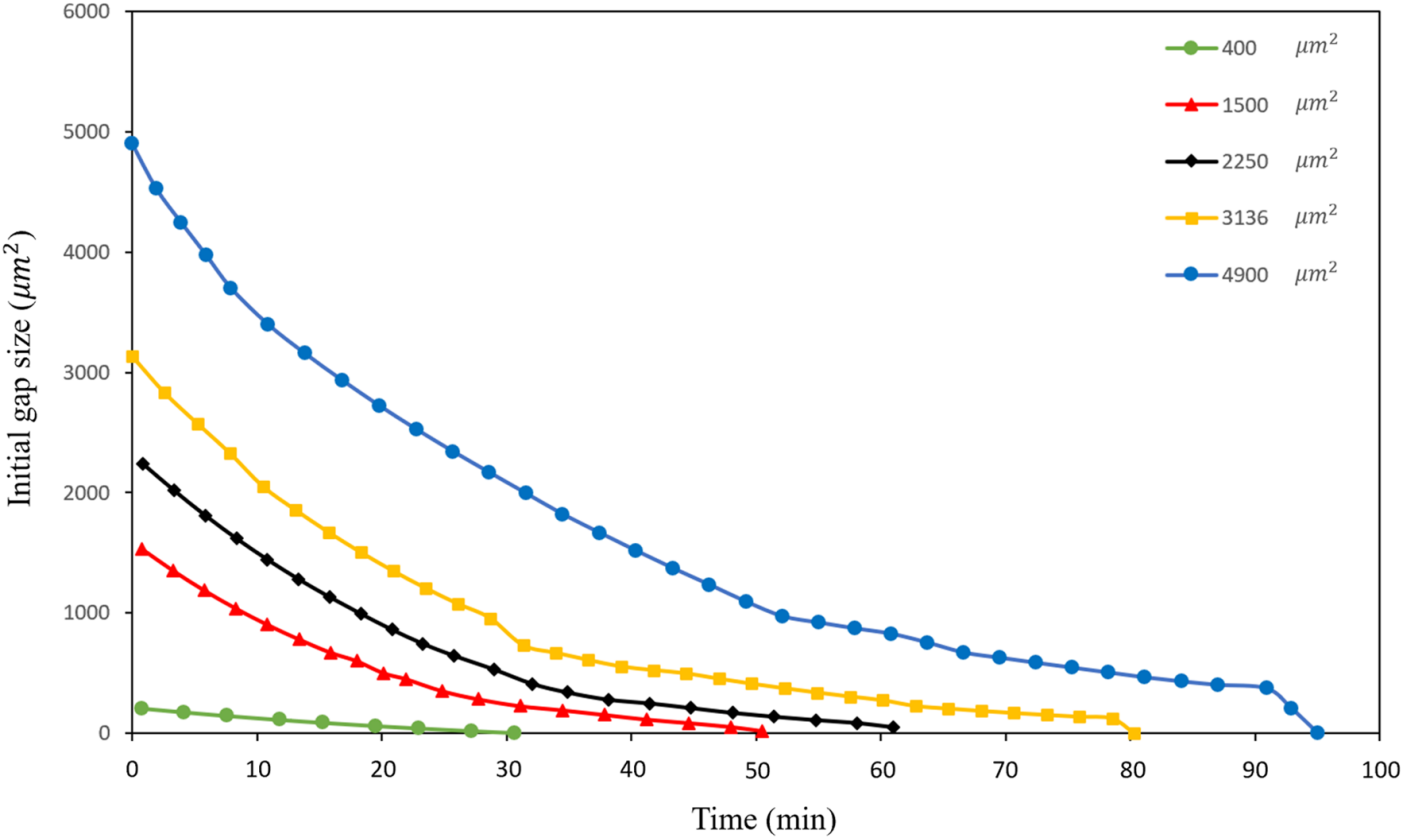
Wound area decreases with time, for different wound sizes ranging from 400 ${\upmu {\text{m}}}^{2}$ to 4900 ${\upmu {\text{m}}}^{2}$.

**Figure 6:**
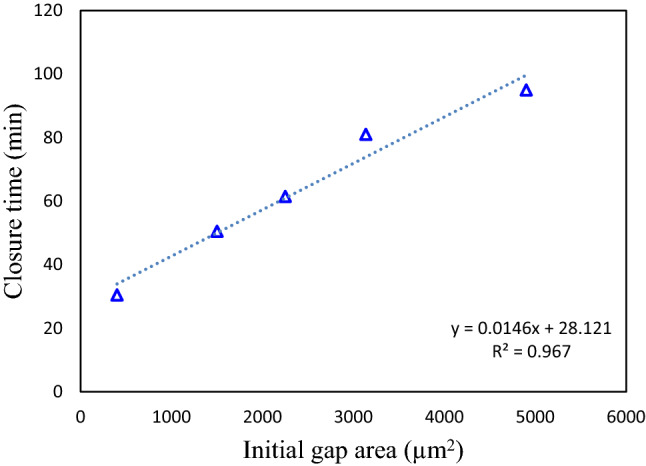
Wound closure time as a function of the initial wound gap area. The linear fitting of simulation data is shown by the dashed line.

Many experimental studies have found there exists a relationship between the original gap size and the wound closure time^2,9,71–73^. McGrath et al.^74^ showed that during the wound closure process, the remaining wound area could be described with a simple exponential dependence on time. Anon et al.^2^ found that during epithelial wound closure, the decrease of the wound area with time was strikingly linear with time down to a complete closure for pillar removal wounds. In order to study the time evolution of the gap area during the epithelial closure, the initial wound area was set in the range from 400 ${\upmu {\text{m}}}^{2}$ to 4900 ${\upmu {\text{m}}}^{2}$ in our simulation. From the simulation results shown in Fig. 5, it can be seen that for all computed cases, the decrease of the area with time was approximately linear with time, especially at the initial stage of wound closure. The trend in the decrease of the wound area as a function of time was similar for the different initial wound sizes, except for the smallest one (for an initial gap area of 400 ${\upmu {\text{m}}}^{2}$). The closure time was found to vary linearly with the initial gap size, as shown in Fig. 6. Both computational results are consistent with the reported experimental results^2,9^.

### Contribution of lamellipodial protrusion on wound closure efficiency

**Figure 7:**
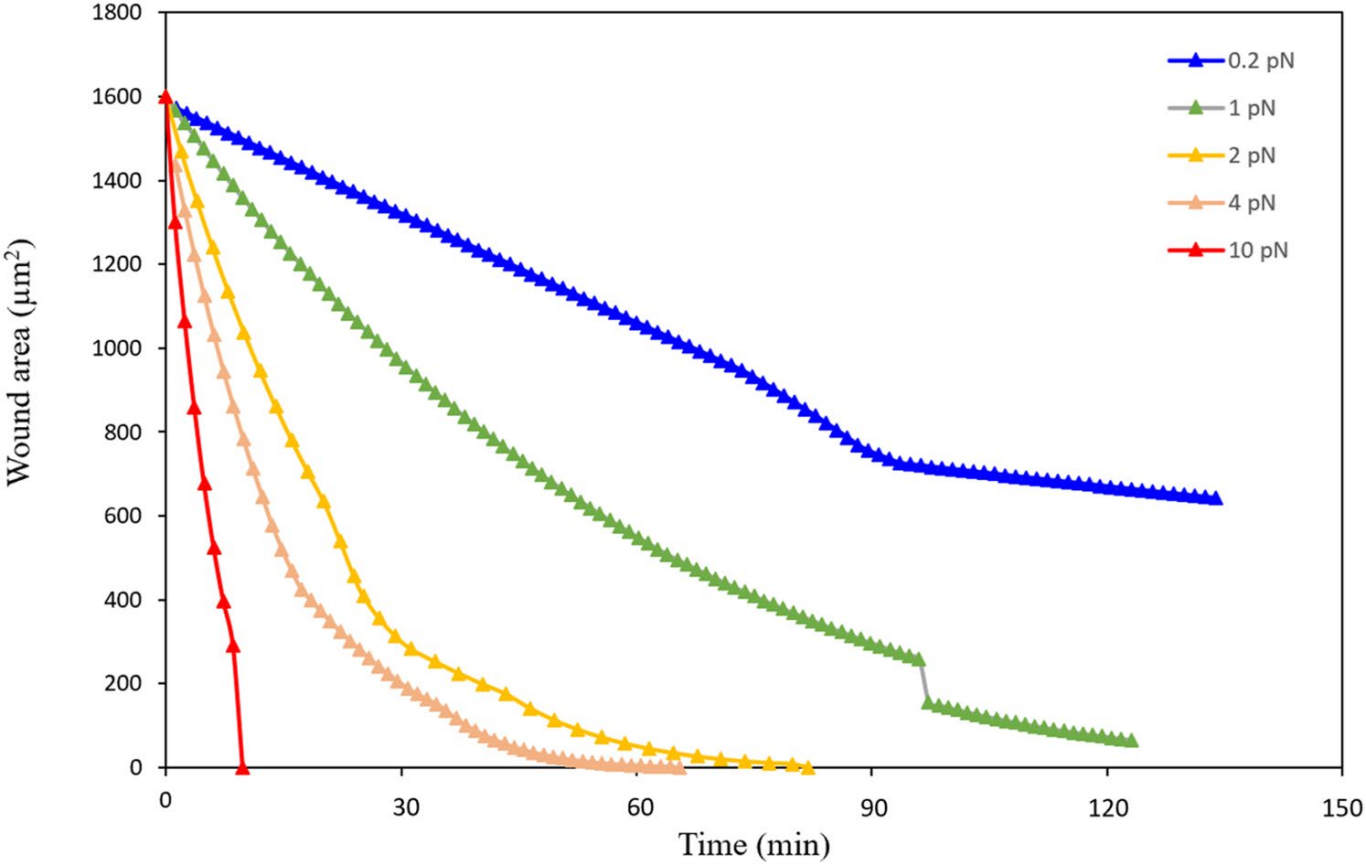
The wound area decreases with time for different protrusion force values ranging from 0.2 to 10 pN.

It has been recognized that cells coordinate the assembly of branched and bundled actin networks to regulate the total mechanical work produced by collective cell motion^75^. In the cell crawling-based wound closure mediated by the lamellipodial protrusion, cells move toward the wound driven by leader cells with the active actin-based protrusions^66^. To study how the lamellipodial protrusion affects the wound closure efficiency during the epithelial closure, we performed case studies with different values of protrusion force ranging from 0.2 to 10 pN in our case studies. From the simulation results shown in Fig. 7, it can be seen that the promotion of lamellipodial protrusion leads to a higher wound closure rate. This result is consistent with the previous experiments^76,77^. We noticed that there is a small jump on the value at the later stage of the wound closure when the force equals 1 pN, which might be attributed to short/sharp edges formed at the later stage of the wound closure process.

## Discussion and conclusion

A 3D continuum physics-based computational model is presented in this study to investigate the epithelial wound closure process. The model employed Voronoi Tessellation to represent the polygonal epithelial cell morphology. The model incorporates the material property of epithelial cells, the cell protrusive force, the intercellular interactions between neighboring cells, and the cell-substrate adhesion between the epithelial cells and the ECM. Through finite element simulation, it is found that the closure efficiency relates to the initial gap size and the intensity of lamellipodial protrusion. Promotion of lamellipodial protrusion was found to result in higher wound closure speed, and the stress was found to be concentrated at the wound edge. It is also shown that cellular normal stress dominates over the cellular shear stresses during the wound closure process. The model presented in this study can be employed as a numerical tool to better understand the complex wound closure mechanism from a mechanical point of view. These results might have the potential to improve effective wound management and optimize the treatment.

Epithelial wound closure is a complex process in which not only biochemical factors, but also physical factors (e.g. cell–cell/substrate adhesion, force of lamellipodia, cell material properties and substrate stiffness, geometry and shape of wound area, and cell division and remodeling) play significant roles in regulating tissue repair. How each individual physical factor will affect the epithelial wound closure is not well understood. Therefore, an in-depth understanding of the various mechanical interactions regulating epithelial wound closure is necessary to gain a better insight into tissue repair. For epithelial wound closure, the particle based model was widely used^78,79^, in which the material properties of cells and intercellular interaction at cell junctions were not modeled explicitly and the model is not able to capture cell stress and deformation, thus the particle based models should be avoided when mechanical cues play an important role^80,81^.

There are several limitations associated with the presented study. Firstly, the epithelial cells were modeled as the simple elastic material which does not consider the detailed cell microstructure and the complex cell material properties. Secondly, the presented computational model does not take into account the factors such as “chemotaxis” or “durotaxis” in cell movements, so the protrusion force applied may not be realistic in real situation. Thirdly, we did not consider any pathological factors in our model. In addition, we didn’t consider the extracellular matrix remodeling during wound healing, which could also alter the simulation results via cell-substrate interactions. Currently, we didn’t study how the geometry and shape of wound area will affect the wound closure. The objective of current research is to establish mechanistic models at cellular level and to develop computational tools for the study of epithelial wound closure. Although it is just a very primitive model, it has the potential to study the role of mechanical interactions on the epithelial wound closure. The current primitive study provides a numerical tool and may open a door for a more advanced study of the epithelial wound closure process.

## Supplementary Information

Supplementary Legends.

Supplementary Movie S1.

## Data Availability

All data generated or analyzed during this study are included in this published article (and its Supplementary files).
