# Supplementary material for: Computational modeling and simulation of epithelial wound closure
Source: Sci Rep. 2023 Apr 17;13:6265. doi: 10.1038/s41598-023-33111-4 (PMC10110613; doi:10.1038/s41598-023-33111-4)
Supplement: Supplementary file 1 — Supplementary Legends. [file 41598_2023_33111_MOESM1_ESM.docx]

Supplementary Information

Supplementary Video S1: Epithelial Wound Closure Process
